# Modeling and Simulation Investigation of Ferroelectric-Based Electrostatic Doping for Tunnelling Field-Effect Transistor

**DOI:** 10.3390/mi14030672

**Published:** 2023-03-17

**Authors:** Dong Wang, Hongxia Liu, Hao Zhang, Ming Cai, Jinfu Lin

**Affiliations:** Key Laboratory for Wide Band Gap Semiconductor Materials and Devices of Education, School of Microelectronics, Xidian University, Xi’an 710071, China

**Keywords:** ferroelectricity, electrostatic doping, tunneling field-effect transistor, TCAD, polarity gate

## Abstract

In this paper, a novel ferroelectric-based electrostatic doping (Fe-ED) nanosheet tunneling field-effect transistor (TFET) is proposed and analyzed using technology computer-aided design (TCAD) Sentaurus simulation software. By inserting a ferroelectric film into the polarity gate, the electrons and holes are induced in an intrinsic silicon film to create the p-source and the n-drain regions, respectively. Device performance is largely independent of the chemical doping profile, potentially freeing it from issues related to abrupt junctions, dopant variability, and solid solubility. An improved ON-state current and I_ON_/I_OFF_ ratio have been demonstrated in a 3D-calibrated simulation, and the Fe-ED NSTFET’s on-state current has increased significantly. According to our study, Fe-ED can be used in versatile reconfigurable nanoscale transistors as well as highly integrated circuits as an effective doping strategy.

## 1. Introduction

Recently, silicon nanosheet devices have attracted a significant increase in interest, both for use in integrated nanoscale electronics and for studying fundamental properties in small scales [1,2,3,4]. In order to achieve reliable devices or test structures at nanoscale dimensions, it is necessary to control doping levels on silicon nanosheets and to reduce leakage power. In comparison to short-channel MOSFETs, engineering of gate work function [5,6], lateral channels [7], multiple gate geometry [8], homodielectric based SOI/SON FETs [9], negative capacitance transistor [10,11], and tunneling transistors [12,13] have demonstrated admirable performance. Due to the band-to-band tunneling (BTBT) mechanism, devices with steep slopes, such as Tunnel-FETs, have reduced leakage current and facilitate further scaling without degradation of performance. Nevertheless, the ultra-sharp doping profile requirements, both in MOSFETs as well as TFETs, require complex fabrication processes. Recently, electrostatic doping (ED) has been used in junctionless field-effect transistors (JLFETs) [14] and junctionless tunnel field-effect transistors (JL-TFETs) [13] to address these issues. To realize Source/Channel/Drain regions in TFETs, the background doping must be modified from (N^+^-N^+^-N^+^)/(P^+^-P^+^-P^+^) to (P^+^-I-N^+^)/(N^+^-I-P^+^) using ED. By using electrostatic-doped junctions, fabrication is easier, less variability exists, and further scaling can be achieved [14,15,16].

An ED approach relies on the relative separation between semiconductor’s energy bands and an adjacent electrode’s Fermi level at close proximity to the interface to control doping. The workfunction engineering approach (WF-ED) and external bias approach (Bias-ED) provide effective controls [17]. Recently, Bias-ED has gained considerable attention due to its switchable functionality, as it produces versatile reconfigurable transistors [18,19,20,21]. Despite this, a continuous bias is required to counteract the volatile nature, which is undesirable. It is critical to investigate the method for attaining ED with non-volatility while maintaining reconfigurability for high-density integration. As ferroelectric materials are nonvolatile and programmable in polarization, they are becoming increasingly attractive for use in applications utilizing nonvolatile memory (NVM), enabling nonvolatility and reconfigurability in ED [22,23,24,25,26].

The Control Gate (CG) in our device operates conventionally by switching the device on and off. Polarity Gate (PG) electrodes act on the side regions of the channel close to the Source/Drain Schottky junction, dynamically switching polarity. In this paper, we propose an electrostatic doping technique based on ferroelectrics, which has the advantages of both nonvolatility and reconfigurability. Afterward, using numerical simulation, we demonstrate that Fe-ED nanosheet TFETs (NSTFETs) can be reconfigured without adding bias, with an ultrahigh doping concentration of 7 × 10^20^ cm^−3^ is promising for refueling CMOS scaling.

## 2. Concept and Methodology

**Figure 1:**
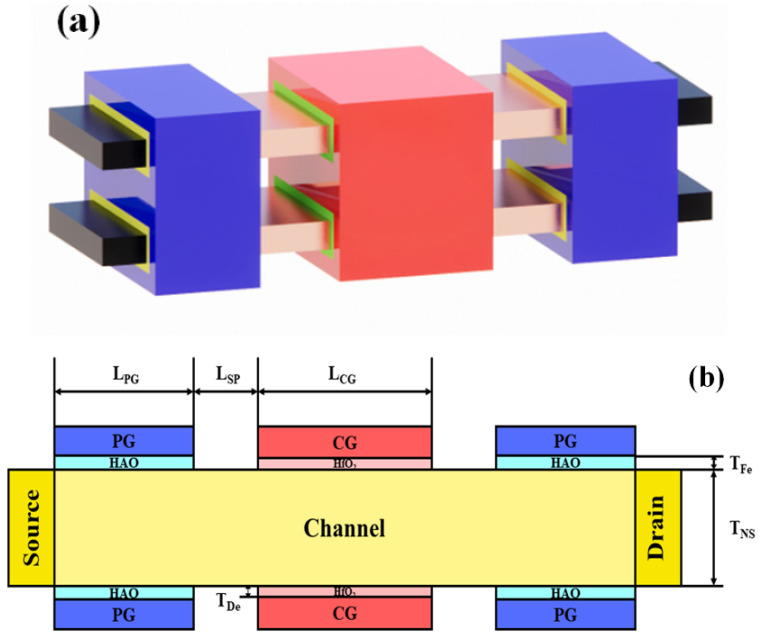
(**a**) Schematic structure and (**b**) cross section of Fe-ED NSTFET.

The three-dimensional (3D) and cross-sectional view of Fe-ED NSTFET are shown in Figure 1a,b. There are two sets of gate electrodes: (a) as a conventional gate, the control gate (CG) switches ON and OFF identically and vice versa; (b) polarity gates (PGs) control the device’s polarity and conduction mechanism. Near the source and drain contacts, they are embedded in the side regions of the channel. It is possible to maintain significant remnant polarization (P_r_) in the polarity gate by inserting the ferroelectric film into it, thus creating nonvolatile and programmable doping within the surrounding source/drain (S/D) regions. After certain pulses, P_r_ is aligned perpendicularly to the channel surface. Simulations were performed at room temperature using Synopsys Sentaurus. During simulation, mobility models such as the Lombardi mobility model, high field saturation model, and Philips Unified mobility are included. The effects of Shockley Read Hall recombination (to take into account recombination via traps), Auger effects, and tunneling via band to band recombination are also enabled.

**Figure 2:**
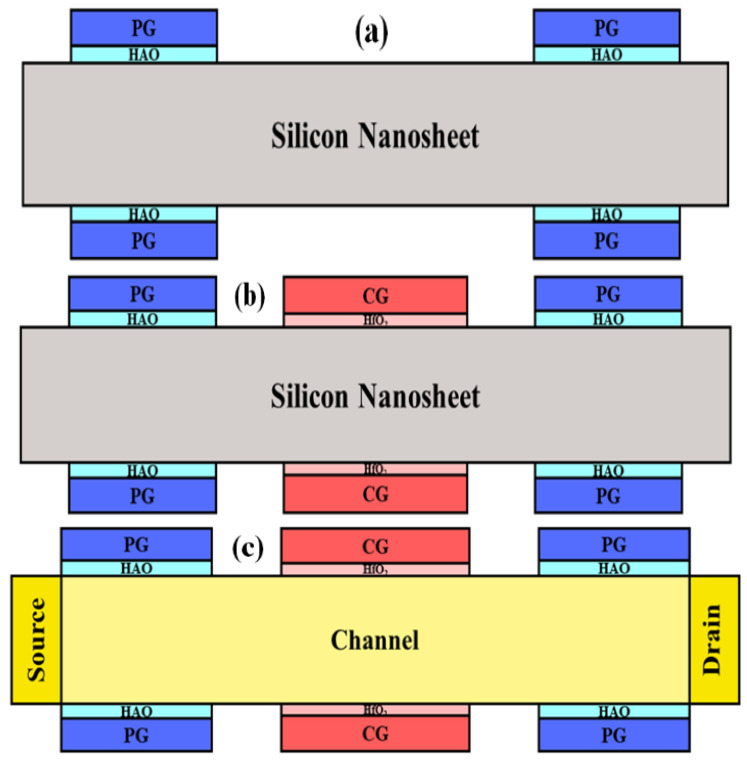
Fe-ED NSTFET fabrication steps: (**a**) polarity gate, (**b**) self-aligned control gate, and (**c**) S/D contacts.

Using the fabrication flow described in [27] for nanowire FETs with gate-all-around electrodes, Fe-ED NSTFETs can be realized. Metal gate/ferroelectric stacks are deposited, patterned, and etched to form polarity gates following the formation of nanosheet structures (Figure 2a). Afterwards, polarity gates with self-aligning control gates (CGs) are manufactured (Figure 2b). Lastly, S/D contacts are established (Figure 2c). Among them, 5 nm-thick Al-doped hafnium oxide (HAO) ferroelectric film is deposited by atomic layer deposition (ALD) at 300 °C using Trimethylaluminum (TMA), Tetrakis(ethylmethylamido)hafnium (TEMAHf), and O_3_ as the reactants [28,29,30]. After that, rapid thermal annealing (RTA) at 800 °C for 30 s is carried out in N_2_ ambient. Due to the hysteresis issue of ferroelectric film, corresponding technique must be applied to avoid it [31].

**Figure 3:**
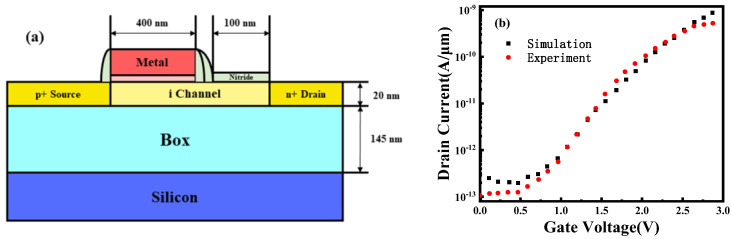
(**a**) Schematic of SOI TFET cell. (**b**) Comparison of simulated and experimental transfer characteristics results for SOI TFET.

Fe-ED NSTFET was simulated by TCAD tools using a non-localized band–band tunneling model. Tunneling model parameters are calibrated from SOI TFET experimental results as shown in Figure 3a. The non-localized band–band tunneling model in Sentaurus uses the Kane model. In the Kane model, the band–band tunneling generation rate is expressed as:

(1) $$R_{net}=A{\left(\frac{F}{F_{0}}\right)}^{P}\exp(-\frac{B}{F_{0}})$$

where *F*_0_ = 1 V/cm, *P* is 2.5 in phonon-assisted indirect bandgap tunneling, and the parameter A and exponential factor B in Equation (1) are expressed as:

(2) $$A=\frac{g{\left(m_{v}m_{c}\right)}^{\frac{3}{2}}(1+2N_{op}){D_{op}}^{2}{\left(qF_{0}\right)}^{\frac{5}{2}}}{2^{\frac{21}{4}}h^{\frac{5}{2}}{m_{r}}^{\frac{5}{4}}P\varepsilon_{op}{\left[E_{g}\left(300K\right)+{∆}_{c}\right]}^{\frac{7}{4}}}$$

(3) $$B=\frac{2^{\frac{7}{2}}\pi {m_{r}}^{\frac{1}{2}}{\left[E_{g}\left(300K\right)+{∆}_{c}\right]}^{\frac{3}{2}}}{3qh}$$

The valence band effective mass m_v_, the conduction band effective mass m_c_, and the reduced tunneling effective mass m_r_ can be expressed as follows:

(4) $$\frac{1}{m_{c}}=\frac{1}{{2m}_{r}}+\frac{1}{m_{0}}$$

(5) $$\frac{1}{m_{v}}=\frac{1}{{2m}_{r}}-\frac{1}{m_{0}}$$

In this paper, the experimental data and model calibration methods are used to calibrate the band–band tunneling model parameters A and B of silicon materials [32]. During model calibration, the model parameters A and B are recalculated by selecting different reduced tunneling effective masses m_r_, and the default parameters of the software are modified to the calculated values. As shown in Figure 3b, it can be seen from the transfer characteristic curve that when the effective mass m_r_ is set to 0.33 m_0_, the simulation and experimental results are the closest. Therefore, the optimal values of the band-to-band tunneling parameters A and B of the silicon material are 1.63 × 10^14^ cm**^−^**^3^ and 1.47 × 10^7^ V/cm, respectively.

## 3. Results and Discussion

### 3.1. The Device Structure of Fe-ED NSTFET

**Figure 4:**
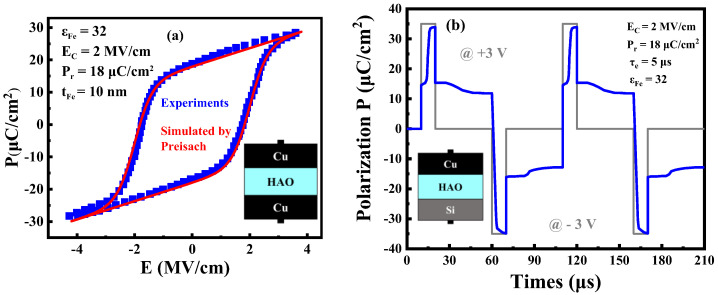
(**a**) Simulated and measured P-E curves, with fitting parameters extracted. (**b**) Simulation of polarity gate’s polarization response to the square waves of ±3 V for 10 µs.

The structural parameters of Fe-ED NSTFET in the simulation are listed in Table 1, where the coercive electric field E_c_, the remanent polarization P_r_, and the dielectric constant ε_Fe_ are parameters of the ferroelectric material in the device. As shown in Figure 4a, Al-doped hafnium oxide ferroelectric material polarization versus electric field curve in the experiment and simulation is well fitted, and the parameters of the ferroelectric material are extracted from the curve [28]. Figure 4b shows a non-volatile polarized charge of +11 μC/cm^2^ remains in the PG under a pulse of +3 V; similarly, a pulsed polarized charge of −3 V to the PG transforms into −11 µC/cm^2^. Finally, the corresponding carriers are generated by the pulse, forming the non-volatilely doping in source and drain regions.

### 3.2. The Operating Mechanism of Fe-ED NSTFET

**Figure 5:**
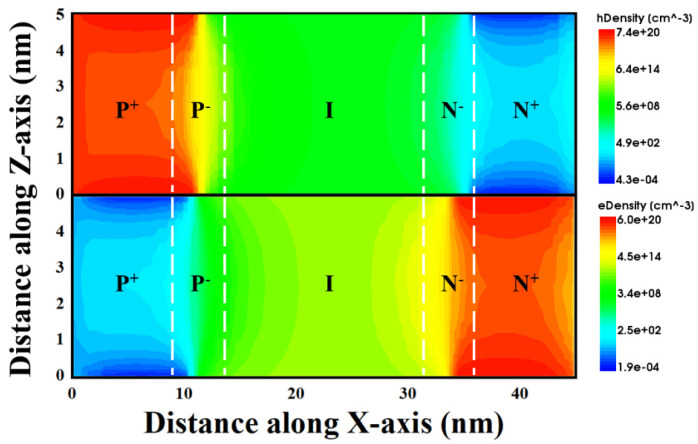
Contour plots of electron and hole carrier density for Fe-ED NSTFETs after pulse, at V_PG_ = V_GS_ = V_DS_ = 0 V, showing nonvolatile and reconfigurable doping.

Using ferroelectric doping to generate nonvolatile source and drain regions, a −3 V pulse is applied to the PG in the source region, and a +3 V pulse is applied to the PG in the drain region, while the other electrodes are all grounded, including the Gate, Source and Drain. Figure 5 shows the electron and hole distributions of the Fe-ED NSTFET, respectively. Obviously, after the ±3 V pulse, the ultrahigh carrier concentration at the source/drain regions has been achieved. Potential barriers are formed between source/drain and channel regions, and therefore, such a p-i-n structure generates a TFET. As can be seen in Figure 5, the electron/hole concentration exceeds 7 × 10^20^ cm^−3^, which results in ultra-low resistance in the source/drain regions. As we all know, the higher the concentration of ion implantation in the device doping process, the more serious the lattice damage in the device, and the ferroelectric doping technology can effectively avoid many problems of chemical doping.

### 3.3. The Transfer and Output Characteristics Analysis

**Figure 6:**
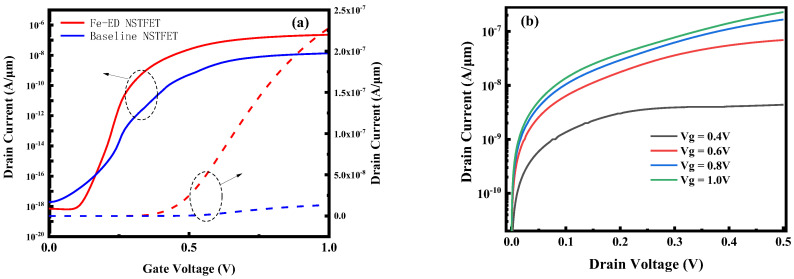
(**a**) ID–VG curves of Fe-ED NSTFETs and baseline devices. (**b**) ID–VD curves of Fe-ED NSTFETs.

Figure 6a,b show the transfer characteristics and output characteristics of Fe-ED NSTFET and baseline TFET. The off-state currents of Fe-ED NSTFET and baseline TFET are 7.1 × 10^−19^ A/µm and 2.0 × 10^−18^ A/µm, respectively, while the on-state currents of the Fe-ED NSTFET and the baseline TFET are 1.4 × 10^−8^ A/µm and 2.3 × 10^−7^ A/µm, and hence, there is a two-order of magnitude improvement in on-state current and switching ratio. Subthreshold swing is an important parameter for evaluating TFETs. When we compare the subthreshold swing of Fe-ED NSTFET and the baseline TFET from 58.7 mV/dec to 28.4 mV/dec. Therefore, Fe-ED NSTFETs have better applications at low voltage and low power.

### 3.4. The Energy Band Analysis of Fe-ED NSTFET

**Figure 7:**
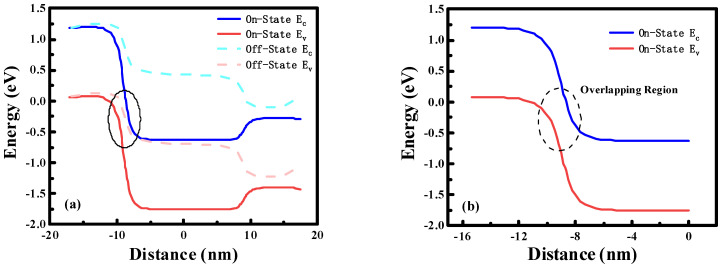
(**a**) Schematic of band diagrams of Fe-ED NSTFETs. (**b**) Energy band diagram at the junction of the source and the channel region.

**Figure 8:**
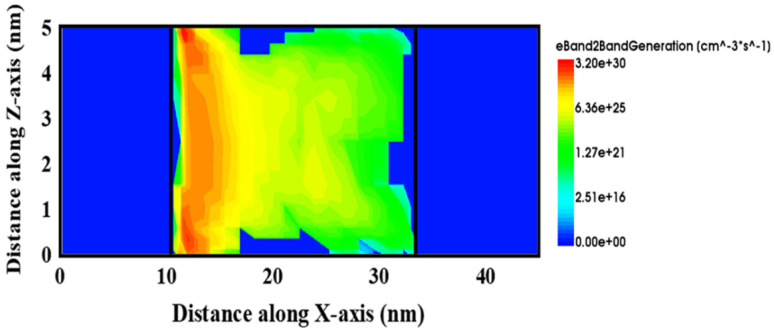
The electron band-to-band generation rate of Fe-ED NSTFET.

The energy band barrier of Fe-ED NSTFETs is the main factor affecting band tunneling, as shown in Figure 7. Figure 7a shows that the conduction bands of the channel region and the valence bands of the source region are not overlapping in the off state. In this case, from the source region to the channel region, electrons cannot pass through the barrier since their energy quantum state is not the same on both sides of the tunneling junction; therefore, there is almost no tunneling current in the off state. To observe the energy band in the on state more clearly, Figure 7b is the energy band diagram at the junction of the source region and the channel. By applying a forward voltage to the gate, the source region’s energy band is dragged down. At this time, it is expected that the valence band in the source region and the conduction band in the channel will overlap, and the tunnel junction will have equal quantum states, electrons can pass through the potential barrier from the source region to the channel region, thereby generating a considerable current. Figure 8 shows the electron band to band tunneling rate distribution of the device in the on state. Obviously, at the interface between the source region and the channel, tunneling rates are highest, even up to 3 × 10^30^ cm^−3^·s^−1^.

### 3.5. Scaling Capability of Fe-ED NSTFET

With the reduction in MOSFET scale, the switching speed, high-frequency performance, density, cost and function of integrated circuits (ICs) have been greatly improved.

**Figure 9:**
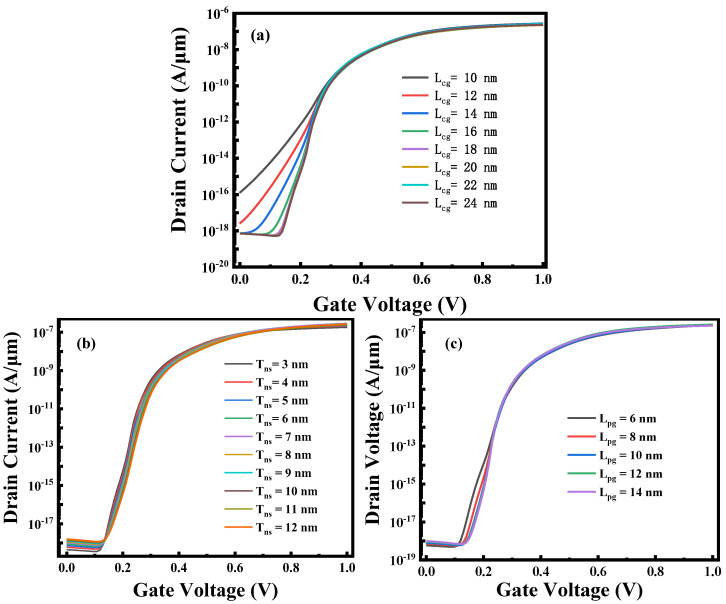
(**a**) Effect of control gate length on the transfer characteristics; (**b**) effect of nanosheet thickness on the transfer characteristics; (**c**) effect of polarity gate length on the transfer characteristics.

Scaling capability has gradually become an indispensable part of measuring the indicators of novel devices. The band tunneling phenomenon of Fe-ED NSTFET is closely related to the device structure parameters, and it is necessary to improve the device performance by optimizing the device parameters. Figure 9a shows the drain current versus gate voltage curve of Fe-ED NSTFET under the different control gate lengths. It can be seen that control gate lengths exceeding 14 nm have little impact on the off-state drain current, and the off-state drain current of Fe-ED NSTFET significantly increases when the control gate length is below 14 nm. With an increasing control gate length, however, the threshold voltage increases, and the device hardly turns on. As shown in Figure 9b, the off-state drain current of Fe-ED NSTFET increases with the increase in nanosheet thickness, and its off-state drain current will decrease accordingly. Therefore, the optimal nanosheet thickness of silicon material is 5 nm in order to select an appropriate on-state and off-state leakage current for Fe-ED NSTFET. Figure 9c demonstrates that the polarity gate length has no significant effect on device performance. Consequently, Fe-ED NSTFET has the scaling capability, especially when the gate length is 14 nm, and the device has favorable transfer characteristics.

## 4. Conclusions

To conclude, we have demonstrated the feasibility of performing Fe-ED TFETs on undoped silicon nanosheets and made them immune to thermal budget concerns. The carrier concentration at the source and drain side was induced through ferroelectric-based electrostatic doping and polarity gate. At various geometries, the proposed device maintains the inherent advantages of the asymmetric p-i-n TFET. Additionally, it demonstrates higher on-state current than conventional p-i-n TFETs of similar geometry. With its high I_ON_/I_OFF_ ratio and simple process, the proposed structure may be advantageous for the development of integrated nanoscale electronics and for studying fundamental properties in small dimensions.

## Figures and Tables

**Table 1:** Device parameters for Fe-ED NSTFET.

Parameters	Values
Remnant Polarization (Pr)	18 µC/cm^2^
Coercive Field (E_C_)	2 MV/cm
Fe Relative Permittivity (ε_Fe_)	32
Thickness of Nanosheet (T_NS_)	5 nm
Width of Nanosheet (W_NS_)	50 nm
Length of Control Gate (L_CG_)	14 nm
Length of Polarity Gate (L_PG_)	8 nm
Length of Spacer (L_SP_)	5 nm
Dielectric EOT (T_DE_)	0.9 nm
Thickness of Fe Layer (T_Fe_)	5 nm
Work Function of Source/Drain (Φ_m_)	4.65 eV

## Data Availability

All data that support the findings of this study are included within the article.
